# scImmOmics: a manually curated resource of single-cell multi-omics immune data

**DOI:** 10.1093/nar/gkae985

**Published:** 2024-11-04

**Authors:** Yan-Yu Li, Li-Wei Zhou, Feng-Cui Qian, Qiao-Li Fang, Zheng-Min Yu, Ting Cui, Fu-Juan Dong, Fu-Hong Cai, Ting-Ting Yu, Li-Dong Li, Qiu-Yu Wang, Yan-Bing Zhu, Hui-Fang Tang, Bao-Yang Hu, Chun-Quan Li

**Affiliations:** The First Affiliated Hospital & National Health Commission Key Laboratory of Birth Defect Research and Prevention, Hengyang Medical School, University of South China, Hengyang, Hunan 421001, China; Hunan Provincial Key Laboratory of Multi-omics and Artificial Intelligence of Cardiovascular Diseases, University of South China, Hengyang, Hunan 421001, China; Key Laboratory of Rare Pediatric Diseases, Ministry of Education, University of South China, Hengyang, Hunan 421001, China; The First Affiliated Hospital, Institute of Cardiovascular Disease, Hengyang Medical School, University of South China, Hengyang, Hunan 421001, China; School of Computer, University of South China, Hengyang, Hunan 421001, China; Insititute of Biochemistry and Molecular Biology, Hengyang Medical College, University of South China, Hengyang, Hunan 421001, China; Clinical Research Center for Myocardial Injury in Hunan Province, Hengyang, Hunan 421001, China; State Key Laboratory of Stem Cell and Reproductive Biology, Institute of Zoology, Chinese Academy of Sciences, Beijing 100101, China; The First Affiliated Hospital & National Health Commission Key Laboratory of Birth Defect Research and Prevention, Hengyang Medical School, University of South China, Hengyang, Hunan 421001, China; Hunan Provincial Key Laboratory of Multi-omics and Artificial Intelligence of Cardiovascular Diseases, University of South China, Hengyang, Hunan 421001, China; Key Laboratory of Rare Pediatric Diseases, Ministry of Education, University of South China, Hengyang, Hunan 421001, China; Insititute of Biochemistry and Molecular Biology, Hengyang Medical College, University of South China, Hengyang, Hunan 421001, China; School of Computer, University of South China, Hengyang, Hunan 421001, China; School of Computer, University of South China, Hengyang, Hunan 421001, China; Insititute of Biochemistry and Molecular Biology, Hengyang Medical College, University of South China, Hengyang, Hunan 421001, China; School of Computer, University of South China, Hengyang, Hunan 421001, China; School of Computer, University of South China, Hengyang, Hunan 421001, China; School of Computer, University of South China, Hengyang, Hunan 421001, China; School of Computer, University of South China, Hengyang, Hunan 421001, China; The First Affiliated Hospital & National Health Commission Key Laboratory of Birth Defect Research and Prevention, Hengyang Medical School, University of South China, Hengyang, Hunan 421001, China; Hunan Provincial Key Laboratory of Multi-omics and Artificial Intelligence of Cardiovascular Diseases, University of South China, Hengyang, Hunan 421001, China; Key Laboratory of Rare Pediatric Diseases, Ministry of Education, University of South China, Hengyang, Hunan 421001, China; Insititute of Biochemistry and Molecular Biology, Hengyang Medical College, University of South China, Hengyang, Hunan 421001, China; Beijing Clinical Research Institute, Beijing Friendship Hospital, Capital Medical University, Beijing 100050, China; The First Affiliated Hospital & National Health Commission Key Laboratory of Birth Defect Research and Prevention, Hengyang Medical School, University of South China, Hengyang, Hunan 421001, China; Hunan Provincial Key Laboratory of Multi-omics and Artificial Intelligence of Cardiovascular Diseases, University of South China, Hengyang, Hunan 421001, China; The First Affiliated Hospital, Institute of Cardiovascular Disease, Hengyang Medical School, University of South China, Hengyang, Hunan 421001, China; Clinical Research Center for Myocardial Injury in Hunan Province, Hengyang, Hunan 421001, China; State Key Laboratory of Stem Cell and Reproductive Biology, Institute of Zoology, Chinese Academy of Sciences, Beijing 100101, China; The First Affiliated Hospital & National Health Commission Key Laboratory of Birth Defect Research and Prevention, Hengyang Medical School, University of South China, Hengyang, Hunan 421001, China; Hunan Provincial Key Laboratory of Multi-omics and Artificial Intelligence of Cardiovascular Diseases, University of South China, Hengyang, Hunan 421001, China; Key Laboratory of Rare Pediatric Diseases, Ministry of Education, University of South China, Hengyang, Hunan 421001, China; The First Affiliated Hospital, Institute of Cardiovascular Disease, Hengyang Medical School, University of South China, Hengyang, Hunan 421001, China; School of Computer, University of South China, Hengyang, Hunan 421001, China; Insititute of Biochemistry and Molecular Biology, Hengyang Medical College, University of South China, Hengyang, Hunan 421001, China; Clinical Research Center for Myocardial Injury in Hunan Province, Hengyang, Hunan 421001, China

## Abstract

Single-cell sequencing technology has enabled the discovery and characterization of subpopulations of immune cells with unique functions, which is critical for revealing immune responses under healthy or disease conditions. Efforts have been made to collect and curate single-cell RNA sequencing (scRNA-seq) data, yet an immune-specific single-cell multi-omics atlas with harmonized metadata is still lacking. Here, we present scImmOmics (https://bio.liclab.net/scImmOmics/home), a manually curated single-cell multi-omics immune database constructed based on high-quality immune cells with known immune cell labels. Currently, scImmOmics documents >2.9 million cell-type labeled immune cells derived from seven single-cell sequencing technologies, involving 131 immune cell types, 47 tissues and 4 species. To ensure data consistency, we standardized the nomenclature of immune cell types and presented them in a hierarchical tree structure to clearly describe the lineage relationships within the immune system. scImmOmics also provides comprehensive immune regulatory information, including T-cell/B-cell receptor sequencing clonotype information, cell-specific regulatory information (e.g. gene/chromatin accessibility/protein/transcription factor states within known cell types, cell-to-cell communication and co-expression networks) and immune cell responses to cytokines. Collectively, scImmOmics is a comprehensive and valuable platform for unraveling the heterogeneity and diversity of immune cells and elucidating the specific regulatory mechanisms at the single-cell level.

## Graphical Abstract

**Graphical Abstract:**
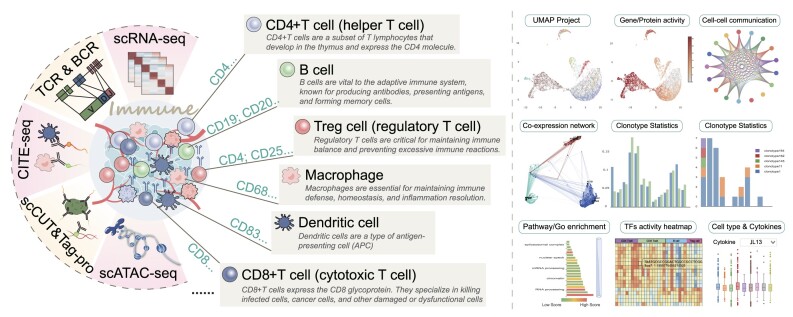

## Introduction

Immune cells, serving as the central component of the immune system, play crucial roles in maintaining immune function, responding to pathogens, and regulating inflammation in diseases and cancers (1,2). Immune cell investigations have emerged as the pivotal entry point for the comprehensive exploration of physiological and pathological states. Extensive evidence indicates that various immune cell subsets are accompanied by complex molecular and cellular transformations, executing specialized functions in different tissue microenvironments (3,4). The rapid development of single-cell immune profiling techniques has enabled an in-depth exploration of the specific subpopulations of immune cells in diverse physiological and pathological states (5–7). For instance, specific macrophages and conventional dendritic cells (cDCs) have been proven as key mediators of cellular cross-talk in the tumor microenvironment based on single-cell analysis (8). However, the high dimensionality of single-cell sequencing data and the complexities of immune cells present challenges in the management and downstream analysis of such data, particularly in terms of cell type annotation, which often demands profound immunological expertise. Researchers have utilized advanced methodologies, such as flow cytometry sorting and manual annotation of immune markers, to precisely categorize immune cells in their publications. Although this approach is labor-intensive, it not only facilitates the establishment of reliable cell types but also unveils a wider array of immune cell subtypes. Accurate annotation of immune cell types is an essential prerequisite for understanding immune cell diversity and functionality. Therefore, it’s an urgent necessity to comprehensively curate and effectively process single-cell immune datasets with known cell labels.

Several immune-related resources have been developed over the past few years, such as SPICA (9), ImmCluster (10), huARdb (11) and JingleBells (12), which were widely used. These databases have provided valuable insights into immune regulatory mechanisms at the single-cell level. Although ImmCluster and SPICA offer single-cell immune data with annotated cell labels, they primarily focus on scRNA-seq, which limits the full mapping of the immune landscape and in-depth exploration of the immune system. The advent of other single-cell multi-omics techniques, such as scTCR-seq, scBCR-seq, scATAC-seq, CITE-seq and scCUT&Tag, has enabled the unlocking of new perspectives for interpreting cellular heterogeneity and investigating the immune system by capturing transcriptomic, epigenetic and functional aspects. For instance, a previous study elucidated the key components, cellular states and clonal relationships of the peripheral and gastrointestinal mucosal immune systems in healthy and ulcerative colitis (UC) samples by integrating single-cell RNA and antigen receptor sequencing (13). Another study drew a detailed atlas of the thymic epithelial cell (TEC) compartment using multi-omics analysis, revealing that medullary TECs contribute to the induction of central tolerance and regulate the homeostasis of other thymus-resident populations (14). These studies enhance our understanding of the immune system’s complexity by combining single-cell data across transcriptomics, epigenomics and proteomics. However, these cells are often scattered across various platforms and publications, highlighting the need to mine and analyze high-quality single-cell multi-omics data for diverse disease scenarios and developmental stages. Furthermore, immune-specific regulatory information, such as immune clonotype and responses to cytokines, will significantly contribute to revealing immune-specific regulatory mechanisms and the functional diversity of immune cell subpopulations. In light of this, the development of a professional and comprehensive immune single-cell multi-omics database with known cell labels is urgently needed.

Here, we developed a manually curated single-cell immune-specific database, scImmOmics (https://bio.liclab.net/scImmOmics/home), which aims to document a large number of available single-cell immune multi-omics data resources, coupled with high-quality cell type annotation and immune-specific regulatory information. The current version of scImmOmics documented >2.9 million cell-type labeled immune cells derived from seven single-cell sequencing technologies (scRNA-seq, scTCR-seq, scBCR-seq, scATAC-seq, CITE-seq, ECCITE-seq and scCUT&Tag-pro), involving 131 immune cell types, 47 tissues and 4 species. Furthermore, scImmOmics provides comprehensive cell/cluster-specific regulatory information, such as clustering, gene expression, differentially expressed genes (DEGs), pathway/Gene Ontology (GO) term/hallmark/immune signatures annotation, differentiation trajectories, cell-to-cell communication, co-expression networks and their signature genes, protein expression, histone modification states, differential chromatin accessibility regions and z-scores of transcription factors (TFs). In particular, scImmOmics offers analyses of immune clonal abundance, immune clonotype distribution across different cell types and immune responses to cytokines, thereby facilitating a deeper understanding of immune cell diversity, clonal expansion, antigen specificity and functional subpopulations. In conclusion, scImmOmics would serve as a valuable platform for users to obtain a more global view of immune cellular heterogeneity and delve into the complex cause-and-effect relationships of the immune system.

## Materials and methods

### Data pre-processing

We queried PubMed with ‘(Single cell) OR (Single-cell sequencing) OR (Single cell epigenomic) OR (scRNA-seq) OR (scTCR-seq) OR (scBCR-seq) OR (scATAC-seq) OR (CITE-seq) OR (ECCITE-seq) OR (scCUT&Tag)’ as keywords and obtained >7000 recent publications. In the literature, the ‘Data Availability’ section usually recorded the resources for data storage, mainly including the NCBI GEO (15), Single Cell Expression Atlas (EMBL-EBI/SCEA) (16), GitHub, Zenodo, Human Cell Atlas Portal (HCA) (17) and CellTypist (18), where the processed files were stored in various formats, such as MatrixMarket, RDS and H5. We first downloaded and retained the data containing metadata, which typically inherited UMAP coordinates and cell/tissue type information from the original articles. Immune cells were extracted and filtered according to cell type information. Then, we manually adjusted and extended the meta-information to include species, tissue, health/disease status, data source, platform, PMID, article name, journal and year. To generate a meticulous hierarchical immune cell structure, we further standardized the diverse meta-information through the following steps: (i) conversion of abbreviated cell types to their full names, (ii) mapping the cell types to the Cell Ontology (19), (iii) adding novel cell subtypes not present in the Cell Ontology to our cell-type hierarchies and (iv) unifying tissue names. Finally, >2.9 million cells across 131 immune cell types obtained from diverse platforms were collected, including 10x Genomics, MARS-seq, Seq-Well, Smart-seq2, inDrop and Microwell-seq.

### Differential gene and functional annotation

Based on the known immune cell types, we used the ‘FindAllMarkers’ function from the *R* package Seurat to identify the DEGs of each cell type (20), and the Wilcoxon test was used to determine the significant P values. Genes with log-transformed fold changes (|logFC| > = 0.25) and *P*-values < 0.01 were considered to be differentially expressed. Next, we performed function enrichment analysis for each DEG set using the *R* package clusterProfiler (21). These GO terms, pathways, hallmarks and immune signatures (from MSigDB) with adjusted *P*-values < 0.05 were considered significantly enriched and visualized using bar charts and bubble charts (22–25).

### Differentiation trajectories

Trajectory analysis aids in understanding the transitions and interactions among various cell types, revealing complex dynamic changes within the immune system. Here, we used the *R* package Monocle3 (26) to infer the trajectory of immune cells and their developmental pseudotime, employing the ‘get_earliest_principal_node’ strategy to set the default root node. The *R* package CytoTRACE2 was employed to assess differentiation potency and cellular states by quantifying gene diversity (27).

### Co-expression modules

We constructed gene co-expression network modules and identified key regulators across different groups using high-dimensional Weighted Gene Co-expression Network Analysis (hdWGCNA) (28). For each module, the ‘ModuleConnectivity’ function was used to compute the eigengene-based connectivity (kME) and highly connected hub genes.

### Cell–cell communications

To evaluate the cell–cell interactions (CCIs) between different immune cell types at the level of signaling pathways, we performed CellChat (29) analyses based on the CellChatDB database. Each ligand-receptor interaction associated with the signaling pathway was assigned a probability value and permutation tests were performed to infer cell-to-cell communication. All the CCIs with *P*-values < 0.01 were visualized in circle plot based on the weight or count.

### Identification and statistics of clonotypes

Mapping the clonotypic landscape of immune cells can help users understand the mechanisms of antigen receptor recognition and functional diversity within the immune system (30,31). Based on CDR3 sequences derived from scTCR-seq or scBCR-seq data, we calculated the clonal frequency for each cell, the proportion of cells with the clonotype within the current cell type and the clonotype distribution across different cell types. Additionally, the *R* package scRepertoire (32) was utilized to visualize the clonal network, and Startrac, derived from (1), was used to produce diversity indices, encompassing clonal expansion, cross-tissue migration and state transition. Finally, scImmOmics provided detailed information on immune cell type, V(D)J gene annotation and CDR3 sequences. Regarding scRNA-seq samples without coupled scTCR-seq or scBCR-seq data, we further downloaded their FASTQ or BAM format files and applied the TRUST algorithm (33) to *de novo* assemble immune receptor repertories, thereby implementing T cell receptor (TCR) and B cell receptor (BCR) reconstruction.

### Differential accessibility regions (DARs) and functional annotation (scATAC-seq)

Investigating differential accessibility regions (DARs) across various cell types is vital for comprehending immune cell heterogeneity and diversity. For scATAC-seq sample, the DARs for each cell type were calculated using the runDA module of scATAC-pro (34), and the assignGene2Peak function was employed to identify the nearest associated genes. The nearest gene was designated as the one closest to the peak center within the 100 kb. Based on the nearest genes of DARs, we performed GO term, pathway, hallmark and immune signature enrichment analyses using the *R* package clusterProfiler to further elucidate the DAR function for each cell type.

### Co-accessibility and gene activity scores (scATAC-seq)

To elucidate the regulatory mechanisms underlying immune cell function and diversity, we utilized Cicero software (35) to calculate co-accessibility scores for pairs of accessible regions based on correlation methods. A threshold of 0.25 was used to determine significant co-accessibility interactions. Subsequently, these co-accessibility regions were linked to nearby genes by considering their proximity to transcription start sites (TSS) and their co-accessibility with the gene promoter region. Finally, the gene activity scores were determined by summing the accessibility of linked regions weighted by their co-accessibility with the gene’s promoter.

### TF/motif enrichment scores and differential TFs (scATAC-seq)

The enriched TFs of the DARs were determined using the *R* package chromVAR (36), with TF motifs sourced from the JASPAR CORE 2022 database (37) and chromVARmotifs (36). Differential TFs/motifs of each cell type were identified using the Wilcoxon test with *P*-values < 0.05. Finally, we visualized the TF/motif z-scores and differential TFs/motifs using scatter plots and heatmaps, respectively.

### Database implementation

The current version of scImmOmics was developed using MySQL 5.7.17 (http://www.mysql.com) and runs on a Linux-based Apache Web server (http://www.apache.org). SpringBoot (https://spring.io/projects/spring-boot) was used for server-side scripting. The interactive interface was designed and built using Bootstrap v5.3.0 (https://v5.bootcss.com) and JQuery v3.7.1 (http://jquery.com). ECharts (https://www.echartsjs.com/) and Plotly (https://plotly.com/javascript/) were used as the graphical visualization framework. We recommend to use a modern web browser that supports the HTML5 standard, such as Firefox, Google Chrome, Safari, Opera or IE 9.0 + for the best display. Furthermore, scImmOmics was powered by a robust infrastructure based on Docker (https://hub.docker.com/), which enables scalability and portability.

### Description of the database

#### Overview of scImmOmics

**Figure 1.**
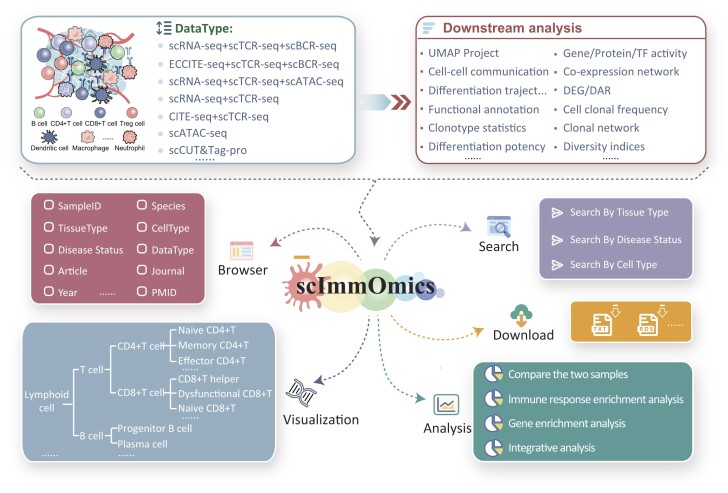
Platform content and construction. scImmOmics manually collected abundant single-cell immune multi-omics data with known cell labels. There are datasets capturing multiple modalities from each cell, including ‘ECCITE-seq + scTCR-seq + scBCR-seq’, ‘scRNA-seq + scTCR-seq + scATAC-seq’, ‘scRNA-seq + scTCR-seq + scBCR-seq’, ‘CITE-seq + scTCR-seq’ and ‘scRNA-seq + scTCR-seq’. For instance, ‘scRNAseq + scTCR-seq + scBCR-seq’ represented the dataset capturing three modalities (scRNA-seq, scTCR-seq and scBCR-seq) from each cell, allowing for a more comprehensive understanding of both transcriptional regulation, immune receptor diversity and epigenetic features within individual cells. scImmOmics supported browsing, searching, analyzing, visualizing and downloading immune-related information.

The main framework and functions of scImmOmics are illustrated in Figure 1. The scImmOmics currently supports immune data with known cell labels derived from multiple single-cell sequencing technologies, involving scRNA-seq, scTCR-seq, scBCR-seq, scATAC-seq, CITE-seq, ECCITE-seq and scCUT&Tag-pro (Supplementary Table S1). There are datasets encompassing multiple modalities, including ‘scRNA-seq + scTCR-seq + scBCR-seq’, ‘ECCITE-seq + scTCR-seq + scBCR-seq’, ‘scRNA-seq + scTCR-seq + scATAC-seq’, ‘CITE-seq + scTCR-seq’ and ‘scRNA-seq + scTCR-seq’. We further performed unified normalization and downstream analysis on these datasets, such as gene/protein/TF activity calculation, DEG identification, pathway/GO term/immune signatures enrichment analysis, cell–cell communication, co-expression network analysis, DAR identification, co-accessibility prediction, immune clonal abundance, immune clonotype distribution across different cell types and immune responses to cytokines. For these datasets and downstream analysis results, scImmOmics supports three search models, two analysis tools and a user-friendly browser and download interface. scImmOmics also offers standardized naming and meticulous hierarchical trees for immune cell types (Figure 1, left bottom panel).

#### Search interface for retrieving scImmOmics datasets

**Figure 2.**
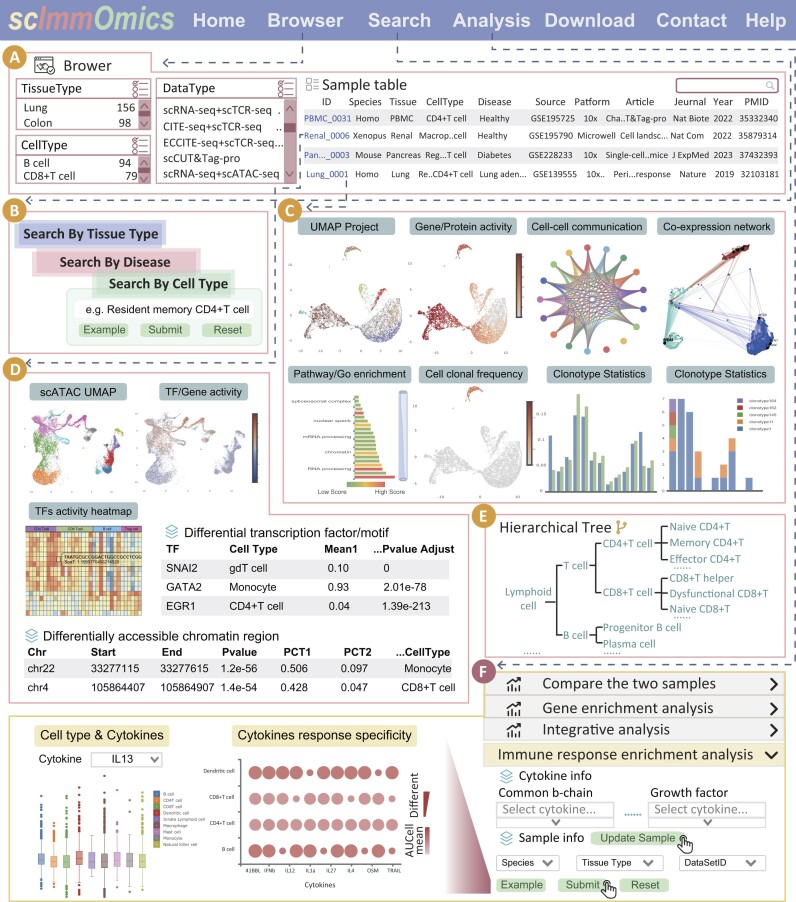
Main functions and usage of scImmOmics. (**A**) The Browse page of scImmOmics. (**B**) Three query modes are provided including ‘Search by Tissue Type’, ‘Search by Disease’ and ‘Search by Cell Type’. (**C**) Detailed information of the scRNA-seq samples. (**D**) Detailed information of the scATAC-seq samples. (**E**) Hierarchical immune cell type trees. (**F**) Four online analysis tools: ‘Compare the two samples’, ‘Gene enrichment analysis’, ‘Integrative analysis’ and ‘Immune response enrichment analysis’.

scImmOmics enables users to search, browse, analyze, visualize and download immune cells of interest (Figure 2). In the ‘Search’ interface, we provide three query methods for searching immune-related samples: ‘Search by Tissue Type’, ’Search by Disease’ and ‘Search by Cell Type’ (Figure 2B). Brief information of the returned samples is displayed in a table on the result page, including the sample ID, source, known cell type, tissue, article name and PMID (Figure 2A). Users can click the sample ID of interest to access the detailed page, where the uniform hierarchical structure of immune cell types involved is highlighted in tree chart (Figure 2E). Regarding scRNA-seq data in the visualization panel, we displayed the UMAP projection colored according to known cell labels, gene expression activity, cell-to-cell communication, co-expression networks, DEGs, function annotation, differentiation potency and pseudo-time (Figure 2C). Meanwhile, the clonal frequency of each immune cell, the ratio of cells with clonotypes across various cell types, and detailed clonotype information for individual immune cells are also displayed (Figure 2C). For the CITE-seq and ECCITE-seq data, we displayed the activity of the proteins of interest in each cell (Figure 2C). For the scATAC-seq data, scImmOmics provides the UMAP projection, activity scores of genes/TFs, TF activity heatmap and table of differential chromatin accessibility regions and TFs (Figure 2D). Additionally, information on both histone modification activity and protein expression measured in the same immune cells can also be displayed on the detailed page of scCUT&Tag-pro.

#### A user-friendly interface for browsing scImmOmics datasets

The browser table describes the information for each sample, including the species, tissue type, cell type, data type, disease status, data source, platform, article, journal, year and PMID (Figure 2A). Users can view datasets and customize filters based on ‘CellType’, ‘TissueType’, ‘DataType’, ‘DiseaseStatus’ and ‘Species’. For example, ‘DiseaseStatus’ filter in the browser interface would help users quickly locate diseases of interest. Users also can filter samples with multiple modalities by selecting the ‘DataType’, including ‘scRNA-seq + scTCR-seq’, ‘scRNA-seq + scATAC-seq’, ‘ECCITE-seq + scTCR-seq + scBCR-seq’, etc. After clicking ‘Sample ID’, the immune cell information for a given dataset can be further viewed.

#### Online analysis tool

We implemented four online tools for immune-related analysis, including ‘Immune response enrichment analysis’, ‘Gene enrichment analysis’, ‘Integrative analysis’ and ‘Compare the two samples’. In the ‘Immune response enrichment analysis’ tool, scImmOmics assesses cytokine activities and immune cell polarization based on gene expression data, given the input of cytokines of interest. Specifically, we obtained the Immune Dictionary of cytokine signatures, which includes DEGs of 1400 cytokine-cell type combinations from (22). The *R* package homologene was utilized for homologous gene switching across species. Using the DEGs of these cytokine signatures, we performed enrichment calculations using the AUCell (38) method on the given data. The analysis results for the AUCell enrichment scores of cytokines of interest in immune cells are displayed using multiple visualization methods, including (i) cell and cytokine enrichment scores, (ii) cell type and cytokine enrichment scores, and (iii) cytokine response specificity across diverse immune cell types (Figure 2F). In the ‘Compare the two samples’ analysis tool, users can select two datasets of interest, and scImmOmics will display detailed information about each dataset. By examining the similarities and differences between the two samples, we can gain deeper insights into immune cell characteristics, clustering patterns and gene expression dynamics across multiple datasets or platforms (Figure 2F). The ‘Gene enrichment analysis’ allowed users to enter a gene set of interest and flexibly assess its correlation with DEGs across diverse immune cell types based on the Hypergeometric test (Figure 2F). The output table contained basic information about identified immune cells (such as SampleID, Species, TissueType, Disease Status, DataType and CellType), *P-*value and adjust *P-*value. The ‘Integrative analysis’ allowed users to select scRNA-seq and scATAC-seq samples of interest, and scImmOmics will display the integrated result and detailed information about each dataset. This function facilitated the exploration of connections across gene expression and regulatory elements, enhancing our understanding of immune cellular states and functions (Figure 2F).

#### Data download

The data in ‘rds’ format and metadata for each sample are available for download on the ‘Download’ page (Figure 2). Users can quickly download data of interest. We also support exporting query results on the ‘Search Result’ page.

### Case study

#### Case study of PBMC dataset

**Figure 3.**
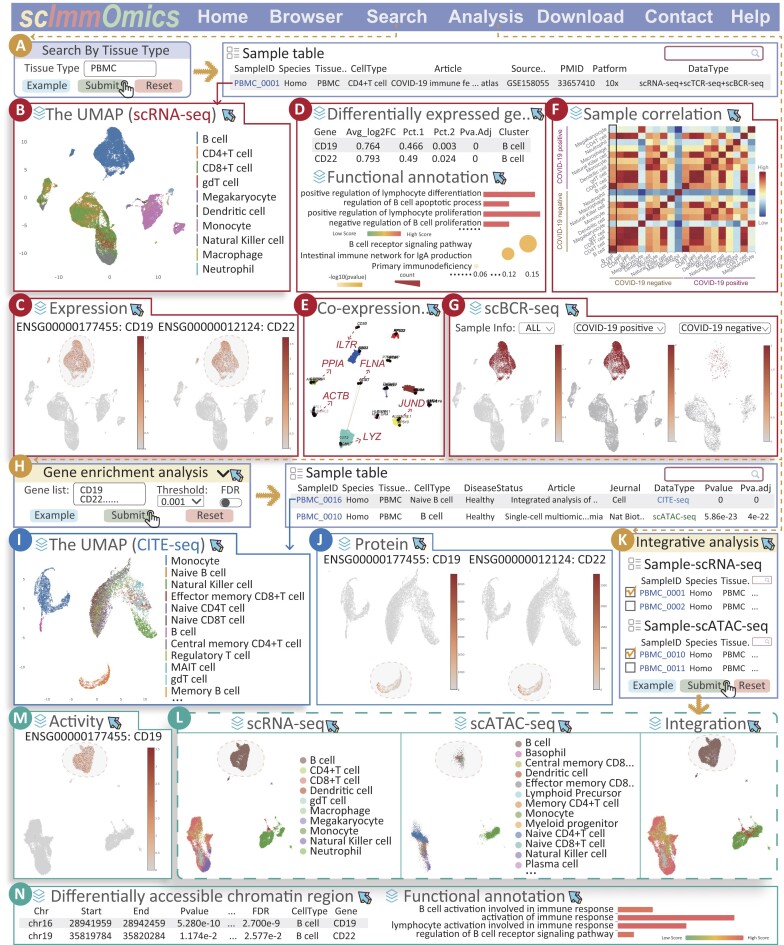
Case study of PBMC dataset. (**A**) The Search page for scImmOmics. (**B**) The UMAP projection of the scRNA-seq sample. (**C**) The gene expression (scRNA-seq). (**D**) Differentially expressed genes and functional annotation (scRNA-seq). (**E**) The co-expression networks (scRNA-seq). (**F**) The sample correlation (scRNA-seq). (**G**) The scBCR-seq information. (**H**) Input and analysis results of the ‘Gene enrichment analysis’ tool. (**I**) The UMAP projection of CITE-seq sample. (**J**) The protein activity (CITE-seq). (**K**) The ‘Integrative analysis’ tool. (**L**) The integrative analysis results of the scRNA-seq sample ‘PBMC_0001’ and the scATAC-seq sample ‘PBMC_0010’, the UMAP projection colored by known cell labels. (**M**) Gene activity in integrative results. (**N**) Differentially accessible chromatin region and functional annotation (scATAC-seq).

To illustrate the usage and advantages of scImmOmics, we input ‘PBMC’ in the search page and selected the sample ‘PBMC_0001’ to access the detailed page (Figure 3A). This sample was labeled ‘scRNA-seq + scTCR-seq + scBCR-seq’, indicating that it contained three distinct types of sequencing data captured from each cell. As shown in the UMAP module, the immune cells were categorized into ten major classes (39) (Figure 3B). We first observed the expression level of known cell markers in the corresponding cell type by clicking the ‘Expression’ module (Figure 3C). Consistent with existing biological knowledge, the B-cell cluster exhibited higher expression levels of CD19 and CD22, both known B-cell markers (40,41). The CD19 and CD22 were then identified as B cell-specific DEGs, indicating the credibility of our downstream analysis (Figure 3D). Function enrichment analysis further confirmed the association of these DEGs with B cells, as they were significantly enriched in B cell-related GO terms and pathways, such as ‘regulation of B-cell apoptotic process’ and ‘B-cell receptor signaling pathway’ (Figure 3D). These findings demonstrated the reliability of the data and analysis outcomes within scImmOmics. Subsequently, several crucial genes were identified within ‘Co-expression modules’ (Figure 3E), including IL7R (42,43), ACTB (44) and JUND (45). Among them, IL-7R has been characterized as a marker of the inflammatory immune response (42,46), suggesting the capability of scImmOmics to delve into the intricate mechanisms of immune regulation. Meanwhile, we could check the correlation of immune cell types across distinct samples by clicking the ‘Sample correlation’ module (Figure 3F). This function allows users to assess the similarity and diversity of immune cells under different conditions. Furthermore, the ‘scBCR-seq’ module also assisted users in exploring the diversity and clonal expansion of B-cell receptors (BCRs) within different biological contexts, shedding light on their roles in immune responses and disease progression (Figure 3G).

To further evaluate the utility of scImmOmics, we used the DEGs of B cell from the ‘PBMC_0001’ sample as input in ‘Gene enrichment analysis’ and set a strict threshold (Figure 3H). The results revealed significant enrichment in B cell-containing samples, highlighting the ability of the scImmOmics platform to capture immune differences and specificity. Notably, we observed significant enrichment in other omics samples, such as the CITE-seq sample ‘PBMC_0016’ and the scATAC-seq sample ‘PBMC_0010’ (Figure 3H). Upon accessing the detailed page by clicking on the sample ID ‘PBMC_0016’, we noticed the high protein activity of CD19 and CD22 in B cell, consistent with the gene expression levels derived from the scRNA-seq sample (Figure 3I and J). This consistency reinforced the reliability of our database, suggesting the feasibility of combining protein activity and gene expression in immune cells. To provide a more comprehensive understanding of the capabilities and advantages of scImmOmics in revealing the complexities of immune regulation, we performed ‘Integrative analysis’ by selecting the abovementioned scRNA-seq sample ‘PBMC_0001’ and scATAC-seq sample ‘PBMC_0010’ (Figure 3K). As shown in Figure 3L, we observed consistent regulatory features in both chromatin accessibility and gene expression data, highlighting the advantages of scImmOmics in combining multiple omics dimensions. In the ‘Gene activity’ module, CD19 also exhibited higher expression and gene activity scores in B cell from scRNA-seq and scATAC-seq samples, respectively (Figure 3M). Here, gene activity scores were quantified using Cicero (35,47), allowed users to further explore the complex regulatory mechanisms driving gene expression (see ‘Materials and methods’ section). By clicking on the ‘differentially accessible chromatin region’ module, we found that both CD19 and CD22 were identified as the nearest genes of differential chromatin accessibility regions (DARs) of B cell (Figure 3N). Meanwhile, these DAR-associated genes were significantly enriched in B cell-related pathways (Figure 3N), reflecting the reliability of the data and robustness in uncovering cell-type-specific immune regulatory features. Overall, these results demonstrated the consistency across epigenomics, transcriptomics and proteomics, highlighting the capability and advantages of scImmOmics in investigating complex immune mechanisms at the multi-omics level.

#### Case study of BoneMarrow dataset

After entering ‘BoneMarrow’ in the search page, bone marrow-related samples were searched out, including the scRNA-seq sample ‘BoneMarrow_0002’ and the scATAC-seq sample ‘BoneMarrow_0011’ (Supplementary Figure S1A and B). We first clicked on the Sample ID ‘BoneMarrow_0002’ of scRNA-seq sample to access its detailed transcriptional level information. In the ‘Expression’ module, we observed the high expression of known markers (CD86, CD33 and CD69) and a key TF (IRF4) within the corresponding cell types (Supplementary Figure S1C and D). These results are consistent with established biological knowledge, providing support for the reliability of subsequent discoveries within scImmOmics (48–58). To showcase the application potential of scImmOmics, we further delved into the transcriptional regulatory mechanisms of immune cells within bone marrow by combining multiple omics dimensions. Specifically, we selected the scATAC-seq sample ‘BoneMarrow_0011’ to access its detailed page (Supplementary Figure S1E). After clicking the ‘Gene activity score’ module, we observed the expected gene activity levels for these markers and TF, consistent with gene expression data derived from scRNA-seq sample (Supplementary Figure S1F). The estimation of gene activity based on the Cicero (35), effectively revealing and deciphering the complex landscape of gene regulation by combining chromatin accessibility within specific cellular contexts.

To clarify the capability of the scImmOmics to capture diverse omics crucial regulatory information, we clicked ‘Co-expression modules’ in the scRNA-seq sample page and found that most hub genes were also identified as DEGs across diverse cell types, such as SRGN, S100A9, HSP90B1, and PRDX4 (Supplementary Figure S1H). Their specific function in the immune system has been reported by literature (59–63), confirming our downstream analysis’s credibility and usefulness. By incorporating chromatin accessibility information (‘Differentially accessible chromatin region’ module on scATAC-seq sample page), we further found multiple DARs were enriched near these hub genes within the corresponding cell type (Supplementary Figure S1I). For instance, S100A9 as the nearest genes of DAR in monocytes, were co-expressed with crucial markers of monocytes, including CD68 (64), CLEC12A (65,66) and CEBPD (67) (Supplementary Figure S1H–I). Among them, the marker CEBPD exhibited higher TF activity by clicking the ‘TF z_score’ module on the scATAC-seq sample page, aligning with its crucial role in this specific cell type (Supplementary Figure S1G–I, see ‘Materials and methods’ section). Both co-expressed genes and DAR associated with S100A9 emphasized its significance in monocyte function, highlighting the reliability and applicability of scImmOmics in dissecting complex immune cell regulatory mechanisms. Furthermore, we noted that POU2F2, as a differential TF motif, did show higher TF activity in mature B cells compared to progenitor B cells, which is consistent with its crucial role in B cell differentiation, particularly in the transition to plasma cells (68) (Supplementary Figure S1G and J). Using the ‘Co-expression modules’ and ‘Differentially expressed genes’ of the scRNA-seq sample page, several POU2F2 co-expressed hub genes, such as HSP90B1 and PRDX4, were further found to be significantly differentially expressed in plasma cells. Overall, scImmOmics consistently captured the characteristics of immune cells across both transcriptional and epigenetic landscapes, offering a comprehensive and integrated view of immune cell function and regulatory mechanisms.

Finally, we performed ‘Immune response enrichment analysis’ on the scRNA-seq sample and found several crucial immune cytokines in the corresponding cell types (Supplementary Figure S1K). For instance, Interleukin-12 (IL-12), Interleukin-23 (IL-23), Interferon-alpha (IFN-α) and Interleukin-1 beta (IL-1β) cytokines showed differential AUCell scores in cDCs, consistent with their established roles in immune regulation and inflammation (69–73). In summary, the capability and biological value of scImmOmics were fully reflected in the abovementioned case results, encompassing diverse aspects such as gene expression, differentially accessible chromatin regions, co-expression, gene activity scores and TF activity.

## Discussion

The advent of single-cell sequencing technology has facilitated the understanding of immune differentiation and activation processes, as well as the heterogeneity of immune cell types. Recent studies have developed several immune-related databases to provide opportunities for analyzing the immune microenvironments at the single-cell level, such as ImmCluster (10), JingleBells (12), SPICA (9) and huARdb (11). While these existing databases promoted the research on the immune system, there are several limitations, especially in terms of multi-omics data integration, manual cell type annotation, a broader range of immune cell subtypes, abundant regulatory information and immune-specific function analyses. Among the existing resources, huARdb and JingleBells use computational software to automatically predict cell types with low coverage. ImmCluster and SPICA offer single-cell immune data with known cell labels, but they primarily focus on scRNA-seq. The above limitations have prompted us to establish scImmOmics, a comprehensive resource of high-quality immune single-cell multi-omics data with known cell labels and comprehensive regulatory analyses.

The scImmOmics is not only a user-friendly database to query, browse and visualize information associated with immune cells but also has multiple highlights and advantages compared to the existing databases as follows: (i) scImmOmics supports immune data derived from multiple single-cell sequencing technologies, involving scRNA-seq, scTCR-seq, scBCR-seq, scATAC-seq, CITE-seq, ECCITE-seq and scCUT&Tag-pro. Integrating more comprehensive experiment technique types will provide new perspectives for interpreting cellular heterogeneity and investigating the immune system; (ii) The current version of scImmOmics covers 131 immune cell types and 46 various tissues, representing a broad range of immune cell and tissue types; (iii) The immune cells cataloged in scImmOmics have been meticulously curated and manually annotated, drawing from original articles; (iv) scImmOmics has a meticulous hierarchical immune cell structure, which offers uniform naming and hierarchical trees of immune cell types; (v) scImmOmics provides a series of immune-related analyses for users, such as immune responses to cytokines, clonotype information identification, function annotation (GO term, pathway and immunologic signature genes), differentiation trajectories, cell-to-cell communication and co-expression networks, and chromatin accessibility/protein/TF/histone modification states.

In summary, scImmOmics is a comprehensive manually curated resource for single-cell immune data. The current version of scImmOmics documents over 2.9 million cell-type labeled immune cells derived from seven single-cell sequencing technologies, involving 131 immune cell types and four species. In the future, we will commit to continuously maintaining and optimizing the database through regular data updates to incorporate the latest available and high-quality single-cell sequencing datasets. By covering a broader range of immune cell types, species, disease states, sequencing technologies and platforms to enhance the utility of our database. We also plan to expand the scope of scImmOmics by incorporating additional immune omics data, such as scChIP-seq and spatial transcriptomics, to enable more comprehensive and multidimensional analyses. Furthermore, we will continue to explore more efficient analysis tools to enhance the speed and usability of data analysis, thereby increasing the overall application value of scImmOmics. Ultimately, we are confident that scImmOmics will emerge as a useful and high-quality platform for exploring the potential functions and regulation of immune cells in diseases and various biological processes.

## Supplementary Material

gkae985_Supplemental_File

## Data Availability

scImmOmics is freely available online at https://bio.liclab.net/scImmOmics/home, and there is no login requirement. The detailed codes are accessible at https://github.com/chunquanlipathway/scImmOmics and https://doi.org/10.5281/zenodo.13918587.
