# Supplementary material for: scImmOmics: a manually curated resource of single-cell multi-omics immune data
Source: Nucleic Acids Res. 2024 Nov 4;53(D1):D1162–72. doi: 10.1093/nar/gkae985 (PMC11701750; doi:10.1093/nar/gkae985)
Supplement: gkae985_Supplemental_File [file gkae985_supplemental_file.pdf]

Table S1. Comparison with other immune-related Databases

Content		scImmOmics	ImmCluster	SPICA	huARdb	JingleBells
Data types	scRNA-seq	√	√	√	√	√
	scTCR/BCR-seq	√			√	
	scATAC-seq	√				
	CITE-seq	√				
	ECCITE-seq	√				
	scCUT&Tag-pro	√				
Immune cell types number		131	61	48	-	-
Known immune cell label		√	√	√		
Hierarchical tree of immune cell types		√				
Immune responses analysis to cytokines		√				
Multi-modal data Integrative analysis		√				
TCR/BCR clonotype information predicted by TRUST4		√				
Clonal network and Startrac diversity indices		√				
Function annotation	GO terms	√	√			
	Pathways	√	√			
	Immune signatures	√	√			
	Hallmark	√	√			
Pseudo time		√				
Cell differentiation potency		√				
Cell to cell communication		√	√			
Co-expression networks		√				
TF activity		√				
Differentially chromatin accessibility regions		√				
Protein activity		√				
Histone modification state		√				

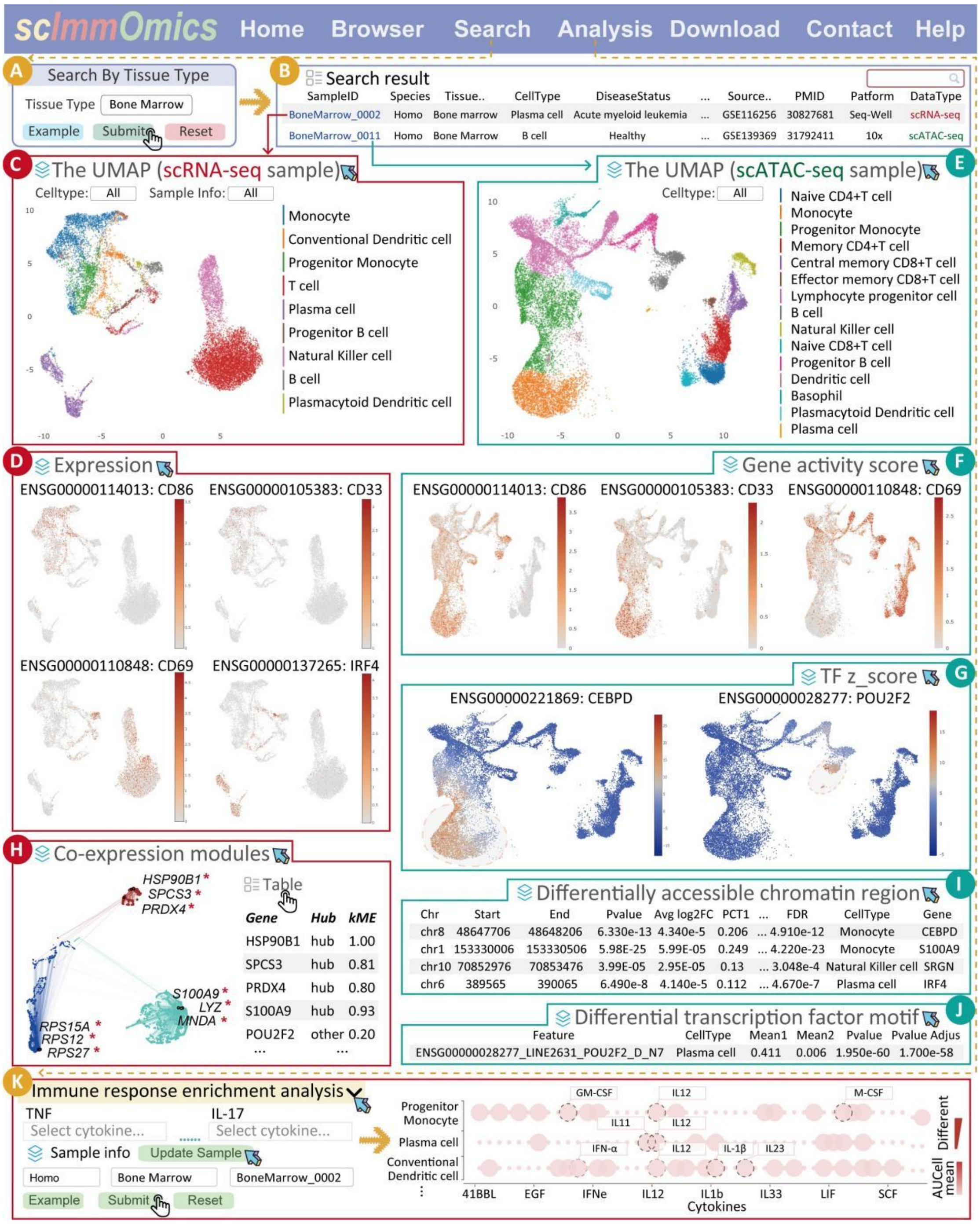

Figure S1. Case study of BoneMarrow dataset. (A) The Search page of scImmOmics. (B) BoneMarrow related sample information, including the scRNA-seq sample ‘BoneMarrow_0002’ and the scATAC-seq sample ‘BoneMarrow_0011’. (C) The UMAP projection colored by known cell labels (scRNA-seq). (D) The gene expression of known markers (scRNA-seq). (E) The UMAP projection of the scATAC-seq sample. (F) Gene activity scores calculated by Cicero (scATAC-seq). (G) The TF activity scores calculated by chromVAR (scATAC-seq). (H) The co-expression

networks, *marked for significant difference (scRNA-seq). (I-J) The table of differential chromatin accessibility region and transcription factor motif (scATAC-seq). (K) Result for ‘Immune response enrichment analysis’. The size of the points represents cell type differences calculated by AUCell scores.
